# Classic and Current Opinions in Human Organ and Tissue Transplantation

**DOI:** 10.7759/cureus.30982

**Published:** 2022-11-01

**Authors:** Angus N Oli, Adekunle Babajide Rowaiye, Samson Adedeji Adejumo, Francis Ifeanyi Anazodo, Rahnuma Ahmad, Susmita Sinha, Mainul Haque, Nihad Adnan

**Affiliations:** 1 Infectious Disease, Nnamdi Azikiwe University, Agulu, NGA; 2 Biotechnology, National Biotechnology Development Agency, Abuja, NGA; 3 Allergy and Immunology, Federal University Oye Ekiti, Oye-Ekiti, NGA; 4 Infectious Disease, Madonna University, Elele, NGA; 5 Physiology, Medical College for Women and Hospital, Dhaka, BGD; 6 Physiology, Khulna City Medical College and Hospital, Khulna, BGD; 7 Pharmacology and Therapeutics, National Defence University of Malaysia, Kuala Lumpur, MYS; 8 Microbiology, Jahangirnagar University, Dhaka, BGD

**Keywords:** allo-transplant, xeno-transplant, types of transplantation, kidney transplantation, brief history, tissue engineering, regenerative medicine, graft tolerance, grafting, tissue transplantation

## Abstract

Graft tolerance is a pathophysiological condition heavily reliant on the dynamic interaction of the innate and adaptive immune systems. Genetic polymorphism determines immune responses to tissue/organ transplantation, and intricate humoral and cell-mediated mechanisms control these responses. In transplantation, the clinician's goal is to achieve a delicate equilibrium between the allogeneic immune response, undesired effects of the immunosuppressive drugs, and the existing morbidities that are potentially life-threatening. Transplant immunopathology involves sensitization, effector, and apoptosis phases which recruit and engages immunological cells like natural killer cells, lymphocytes, neutrophils, and monocytes. Similarly, these cells are involved in the transfer of normal or genetically engineered T cells. Advances in tissue transplantation would involve a profound knowledge of the molecular mechanisms that underpin the respective immunopathology involved and the design of precision medicines that are safe and effective.

## Introduction and background

Transplantation (or grafting) is a surgical or medical procedure involving grafting cells, tissues, or organs from one body part to another, thereby substituting or repairing the damaged, missing, or diseased cells, tissues/or organs. Therefore, a transplant (or a graft) is a group of cells, tissue, or organ grafted into a recipient. Transplants can save lives or restore function to a better quality of life for sick people with vital organ failure if correctly done [1,2], but they can also bring untold challenges [3,4]. The demand for organ transplants increases steadily, with the kidney being one of the most transplanted solid organs. The kidney, liver, heart, lung, pancreas, and small bowel were the most transplanted solid organs in 2019 and accounted for the 153,863 transplants recorded [5]. The COVID-19 pandemic caused a decline in transplantation rates in the early periods of the outbreak. Still, the demand for transplants by diseased patients has not waned, thereby pointing to the continued relevance of tissue transplantation in the medical sphere [6].

Religion, societal behavior and beliefs, and medical ethics are challenges to the general acceptance of tissue or organ transplantation [3]. Furthermore, successful transplantation usually depends on the occurrence or absence of rejection [7], while a shortage of appropriate donor organs is still a major limiting factor in transplantation [7,8]. A good understanding of the immunology of transplantation rejection is vital if more advances are made in this field. The favorable manipulation of the immune cells to promote graft tolerance will be advantageous to solving the problem of tissue rejection [8-10].

This review discusses some historical and relevant opinions and the mechanisms and immunology involved in tissue transplantation and graft rejection.

Materials and methods

The relevant works of literature were obtained by screening online databases, namely: Medline/PubMed, Google Scholar, Scopus, Web of Science, ProQuest, and grey works of literature, using some keyword combinations such as: "Tissue Transplant", "Graft", "Tumor immune response", "Graft tolerance", History of Transplantation", "Types of Transplantation", "Immunology of Transplantation", "Transplant Rejection", "Preventing Rejection" and "Regenerative Medication". Only English publications were included. Also, both original and review articles were used in preparing the study.

## Review

Brief History of Tissue Transplantation

**Figure 1:**
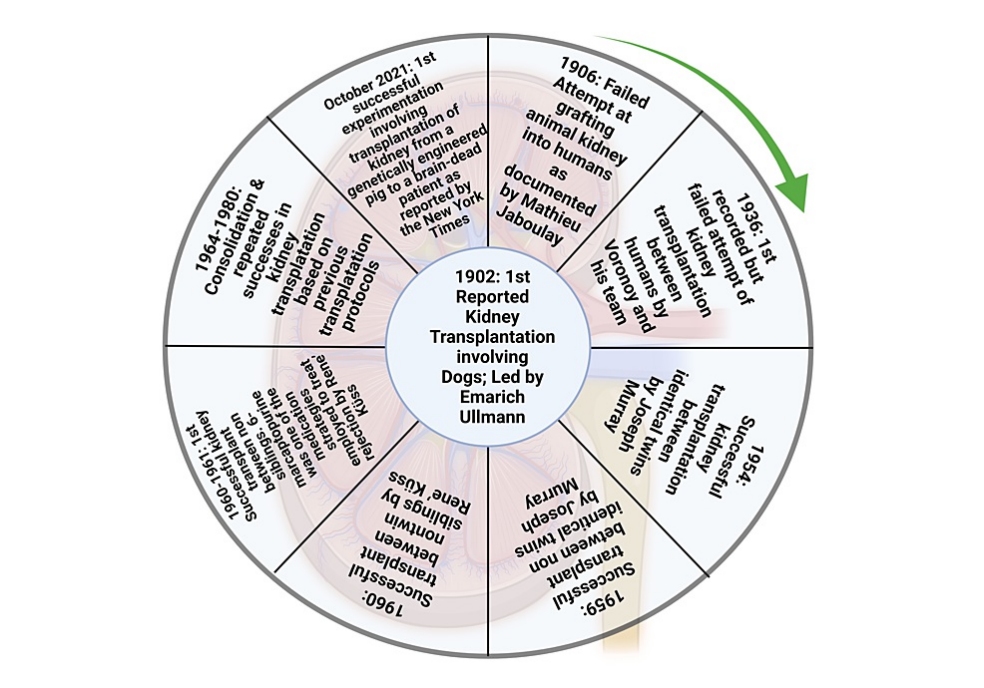
Timeline of landmark achievements in kidney transplantation. This figure has been developed using Biorender [https://biorender.com/] license number: YP24IH1241. Image Credit: Susmita Sinha.

The transfer of tissues and organs, based on needs, among humans is a practice that has its roots in the early centuries. Hamilton [11] narrated extensively how ancient man showed belief in replacing lost organs through procedures of magic and miracles. Hamilton's account is corroborated by documentation on skin transplants done between 3000 and 2500 BC (Before Christ) in India [12]. Early research on tissue transplantation among different species, especially between animals and man and humans, was filled with many challenges despite a few recorded successes [12,13]. Nevertheless, the evolvement of science and better documentation has led to significant progress in the art of transplantation. Alexis Carrel's exploits in vascular science, which involved the transplantation of blood vessels, won him a Nobel Prize and led to better transplantation of other body organs by connecting the arteries and veins of a donor to the corresponding arteries and veins of the recipient [14]. The progress of transplantation up to the current age is better explained by how kidney transplantation has evolved. As recorded by Hakim & Papalois [15], kidney transplants in the early 20^th^ century involved transplantation amongst animals and later from animals to humans. In addition to the first kidney transplant between humans, these transplantation procedures were largely unsuccessful. The failed attempt for the first transplant amongst humans was recorded in Russia in 1936, and a post-mortem donor was involved [16,17]. More attempts at kidney transplantation were later adjudged successful between the 1950s and 1960s. The work progressed from transplants involving identical twins to non-identical twins before climaxing with transplants involving non-siblings. A chronological flow of the significant landmarks [18-20] in kidney transplantation is shown in Figure 1.

Tissue transplantation is now attempted in almost all human body organs; this scientific venture has explored the bones, the eyes, the skin, and solid organs [12, 21]. A most recent account of how transplantation has evolved, especially genetic engineering, has been reported [22]. In what was described as a ground-breaking heart transplant, a male patient received a pig heart that was previously modified genetically. The new heart was said to have performed well for several weeks without rejection before the man eventually died [23]. Although not free of ethical concerns, such attempts at xenotransplantation point to a bright future for the science and art of tissue transplantation.

Types of Transplantation

There are 4 kinds of grafts or transplants (xenograft, isograft, allograft, and autograft) based on the genetic variations between the recipient's and donor's tissues (Table 1). The immunology of grafting is a very complex specialty in medicine [24]. Grafted organs/tissues may either be rejected or destroyed by the recipient's immune system, or the recipients may accept the organ or tissue. If there is rejection, medication to suppress the immunologic response from the recipient is most likely needed. 

**Table 1:** Categories of Organ/Tissue Transplantations with their possible unfavorable results.

S/N	Transplant Type	Donor and Recipient	Potentially unfavorable consequences
1	Xenotransplant	The donor is an animal, while the recipient is human	Rejection is highly possible
2	Allotransplant	The donor and recipient may or may not be relatives but must be same species	Rejection is potentially likely
3	Isotransplant	The donor and recipient are identical twins	Rejection may not be likely
4	Autotransplant	The donor is the self, and the recipient is also self	No envisaged rejection

Xenografting or Xenotransplantation

The word "Xenos" is a Greek word meaning foreign or strange. Xenografting is heterologous transplantation involving the grafting of viable cells, tissues, or organs between two species (e.g., a dog and a pig). It is a cross-species transplantation method. The continued demand for viable organs, tissues, and cells brought about by end-stage organ failure and chronic diseases has been the driving force in this medical/scientific research and practice [25]. However, it has been confronted with the significant challenges of immunological barriers and ethical issues. Organ rejection is widespread in xenotransplantation. In humans, for instance, natural antibodies circulate in the blood, and these cause instant transplant rejection when the organ-donating species is, for example, a pig. Again, the complement systems are often activated each time organs from pigs are grafted into humans or primates and are highly prone to profound system toxicity due to the central role played by the complement system in body homeostasis and metabolism [26]. The porcine complementary proteins are foreign to primate complement regulatory systems. Studies have shown that genetic engineering may be a way out of this complementary system challenge if pigs are genetically modified to contain some human complement regulatory proteins in their cells [27].

Another fundamental challenge facing xenograft is ethical issues. Three ethical issues quickly come to mind when we talk of xenotransplantation: animal rights (effects on the donor animal), human rights (the impact on the human population and the impact on the individual recipient), and interference with nature. An animal rights issue arises because animals, like humans, also have rights to existence and should not be sacrificed in favor of humans [28]. Human rights regarding the recipient can quickly be cleared by obtaining the necessary informed consent. Still, the populace also needs authorization because of the possibility of transferring new pathogens from animal to human populations - a public health risk [1]. The ethical issue of interference with nature may not be so applied. It may be understandable that by interfering with nature, man can free himself from the extinction effects of some natural phenomena [28]. 

A few examples of xenograft include grafting human keratinocytes onto non-human cells (e.g., mice) and then using "ZenSkin" (Reconstructed Human Epidermis) construction as a model for human skin physiology. ZenSkin has applications in pre-clinical and R&D for evaluating how a topical product will affect the human skin [29,30]. Other examples include transfusing non-human blood into human patients and skin grafts from non-humans. Voronoff, in the 1920s, suggested that transplanting slices of chimpanzee testis into geriatric male patients with low sexual vigor would give new energy to such patients [31]. A French Surgeon, Alexis Carrel, developed a method of suturing blood vessels, thereby facilitating organ grafting from non-human primates into human patients [32,33].

Isograft or Isotransplantation

This refers to the inter or intra-transfer of viable tissue(s) or organ(s) between organisms of the same species. Intra-transfer involves the grafting of tissues or organs from a part of the body of an organism to another part of the same organism, while inter-transfer is between separate organisms but of the same species [34].

Corneal transplantation (or keratoplasty), Dacron vascular grafts, and cartilage and bone grafting are all examples of isografts. Renal transplantations are very common and rated as the most successful, primarily because artificial kidney machines are available and the kidney is a paired organ. There is tissue-type compatibility and less risk of fatal organ rejection by the recipient because of donor-recipient matching [35]. A transplant between identical twins is another example of isograft. It is implausible that a recipient will reject an isograft, so an immunosuppressant is unnecessary.

Isograft is an allograft of tissue transplanted between genetically identical individuals of the same species. It refers to tissue grafted from genetically similar twins to another within a species. Autograft transplantation (or autologous grafting) is the grafting of tissue/organs from one area to another position in the same individual patient.

Autograft

Autologous grafting is the transplantation of viable cells, tissue, or organs from one area to another of the same individual or patient. It is frequently referred to as the "gold standard" in bone grafting due to its dependability [36]. The high success rate is due to the fact that bone autograft is a living tissue that contains osteogenic cells and growth factors needed for healing and bone regeneration [37]. Autograft mostly involves tissue transplant where occasionally tissues more desperately elsewhere are required (examples include skin grafts where a skin tissue can be removed from a part of the body with surplus or less important area and transplanted to another area where the tissue is, vein extraction for CABG, etc.) can be extracted and transferred to another part of the same individual. Sometimes an autograft is done to remove the tissue and then treat it in-vitro or treating the person before returning it to the site of action [38]. Other common types of autografting include the reconstruction of the damaged anterior cruciate ligament, skin grafting used to replace damaged or lost skin, and blood vessel grafting used in heart bypass surgery to create an alternative route for blood flow to bypass a blocked coronary artery [39-41]. Autografts pose no risk of disease transmission or immune rejection. However, they have several limitations, which include a limited supply, surgical complications, donor-site pain, and high donor-site morbidity at the procurement site [42].

Allograft

Allografts are tissues such as bone, skin, tendon, ligaments, and heart valves recovered from a human donor who is not an identical twin for transplantation into another person [43]. The transplant is called an allogeneic transplant (allograft) or homograft. Most human organ transplants are under allografting, where an organ is extracted from an individual (donor) and transferred to another individual (recipient). Due to the difference in genetic constituents of donor and recipient, allograft may result in a significant immune response that may trigger graft rejection [44]. Allografts have been successfully used in various medical procedures, especially when an autograft cannot be used. Allograft skin is beneficial in patients with burns that cover a large area of the body. It can be used as a temporary dressing while awaiting the healing of autograft donor sites between harvesting sessions [45]. Also, allografts are used in corneal transplantation when a patient has damaged or failed corneas [46].

Pretransplantation screening of allografts is performed to confirm the donor's tissue viability and the donor's health status to eliminate transmissible diseases such as HIV, Syphilis, hepatitis B, and hepatitis C [47]. To ensure the recipient's safety, the allograft is cleaned and aseptically processed using alcohol, antibiotics, and detergents to rid the tissue of as many cellular elements as possible. Chemical sterilization and electromagnetic radiation are also used to destroy microbes [48]. Unlike the autograft, it takes longer to incorporate into the recipient's body. Chronic rejection and toxicity of immunosuppressive drugs used to improve successful allograft acceptance are some challenges facing the clinical execution of allograft transplants [49].

An example of allografting rejection includes transplanting an organ, such as skin, between two parties who are not identical twins. Skin allografts are used for patients with widespread burns or other conditions causing such huge skin loss that the patient does not have enough intact skin to provide the graft. Skin allografts are eventually rejected due to T cell allorecognition leading to an inflammatory immune response. Still, the resultant wounded areas that are evident by the loss of epidermis, caused by prolonged moisture and friction, develop into well-vascularized granules that autografts from the patient have healed sites take readily [50]. However, an example of allografting without organ rejection is a cornea transplant. Cornea transplants are often not rejected because the cornea has no blood vessels resulting in the inability of the host immune system to recognize and reject the graft [51].

Immunology of Transplantation Rejection

Organ rejection is known to result from the interactions between the adaptive and innate immune systems with the implicated lymphocytes, macrophages, neutrophils, and natural killer cells [7]. The histocompatibility antigens (HCA), encoded by histocompatibility genes (HCG), are implicated in the rejection of grafted tissues and organs [52]. Over 40 loci on the HCG are known to encode HCA. However, the loci on the major histocompatibility complex (MHC) have been remarkable for the most dangerous allograft rejection reactions [53]. The human MHC is found on the short arm of chromosome number 6, very close to the complement genes [54]. However, other antigens causing weaker reactions may exhibit strong rejection reactions in combination. An individual can manifest the MHC genes from both allelic pairs on the body cell surface, with each team coming from each parent.

Each child is half identical to the mother and the father regarding the MHC complex. Therefore, it follows that an individual has a 25% likelihood of having a sibling with a similar MHC. This forms the basis of allograft between relatives.

The human MHC genes complex encode-3 prominent Class I alleles, namely human leukocyte antigens (HLA)-A, HLA-B, and HLA-C, and 3 top-class II alleles, HLA-DR, HLA-DQ, and HLA-DP. The occurrence of two or more distinct forms (alternative phenotypes) of HLA-A, HLA-B, or HLA-DR loci is a known cause of failed transplantation. Closely HLA-matched transplant will most unlikely be recognizable and rejectable, and HLA mismatching has grave effects on the recipient's transplant survival [55].

The MHC molecules are classified as either Class I or Class II molecules. While class I molecules reside in cells with a nucleus, class II molecules reside in professional antigen-presenting cells (APCs) [56]. Physiologically, MHC molecules display antigenic peptides on the T cells, and t lymphocytes can only respond to processed and presented antigens that have complexed with the MHC molecules. The class I molecules offer antigenic peptides from within the cell (endogenous- and auto-antigens) to the cluster of differentiation (CD) 8 T cells [critical subpopulation of major histocompatibility complex (MHC) class I-restricted T cell]. Such antigens include intracellular bacteria, viruses, parasites, cancer cells, and self-antigens. The class II molecules process and present exogenous (extracellular) antigens like extracellular bacteria to CD4 T cells [57,58].

Clinical Stages of Graft Rejection

**Figure 2:**
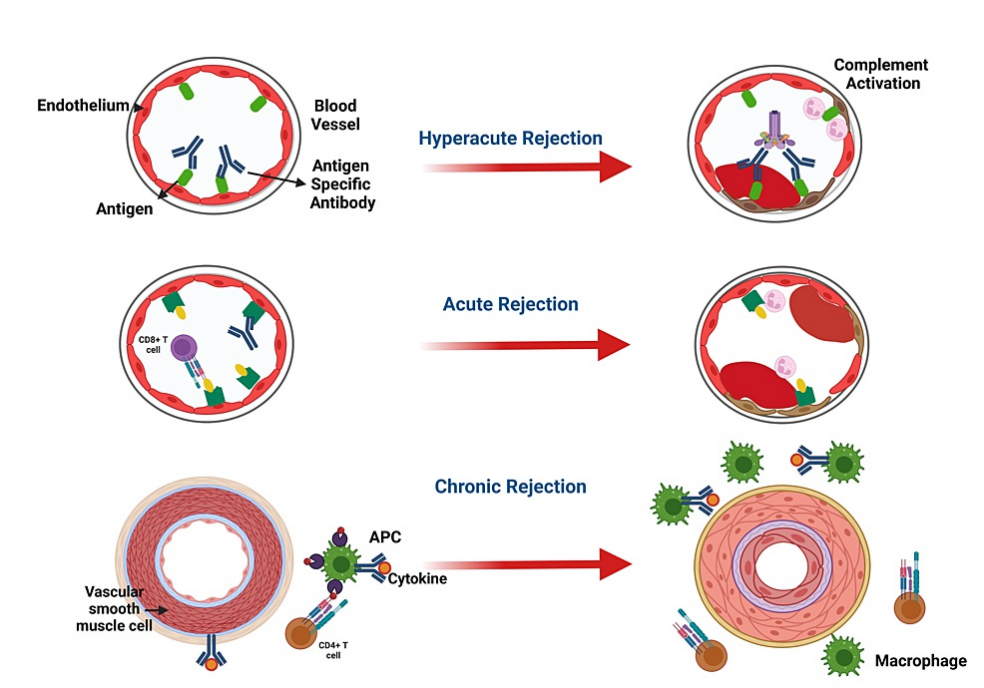
Clinical Stages of Graft Rejection. Notes: APC=Antigen Presenting Cell, CD=Clusters of Differentiation. This figure has been developed using Biorender [https://biorender.com/] License Number: DA24ILUA8K. Image Credit: Susmita Sinha.

The clinical stages involved in graft rejections are summarized in Figure 2.

Hyperacute Rejection 

Hyperacute rejection appears within 24 hours after grafting and only in grafts with profound blood vessels such as the kidney. It is characterized by blood clots inside the blood vessels and graft necrosis. This kind of immunological response is mediated by humoral immunity; the recipient has pre-formed antibodies against the transplant [59,60]. The antigen-antibody complexes cause the stimulation of the complement system, leading to profound clot formation in the capillaries and consequent death of the graft. The liver is relatively more resistant to hyperactive rejection than the kidney, possibly due to dual blood supply to the hepatic system. Proper ABO cross-matching with the exclusion of anti-donor human leukocyte antigen (HLA) antibodies mitigates hyperacute rejection [53]. 

Acute Transplantation Rejection 

Occur any time from the first week to 6 months after the transplant as acute cellular rejection or as acute humoral rejection.

Acute Cellular Rejection 

This is an immunological response in the host's/recipient's lymphoid tissues due to lymphocytes stimulated against donor antigens. The donor's dendritic cells enter the recipient's systemic circulation to function as antigen-presenting cells (APCs) [50,61]. It is common in renal grafts. Acute cellular rejection detection involves biopsy, B-lymphocyte antigen CD20 staining in cases not responding to treatment, negative kidney C4d staining, positive activating lymphocyte markers test, and proteomic study [62]. The first rejection instance is treated with pulse intravenous steroids and may be repeated in cases of recurring or refractory rejections. The second line of treatment (Thymoglobulin and a murine monoclonal antibody, OKT3) may be used for deteriorating grafts. The prognosis depends on the number of rejection episodes, potent drugs, time of rejection from transplantation, and response to treatment [62].

Acute Humoral Rejection 

This is also called acute vascular rejection. It is a severe organ transplant injury mediated by antibodies and complement. The antibodies may be pre-existing or represent anti-donor antibodies developing shortly after grafting [63,64]. Willicombe et al. [65] demonstrated that even low donor-specific antibodies titer not detectable with flow cytometry or complement-dependent cytotoxic cross-matches is linked to lower-ranking renal allograft outcomes. Such patients will likely need augmented immunosuppression. Loupy et al. [66] posited a significant swing in the first-year post-graft in the C4d Banff scores, thus proving the humoral process's changing and painless nature of C4d is not a sufficiently sensitive marker. Still, inflammations in the microvessels and spotting of donor-specific antibodies are better markers of humoral rejection. 

Chronic Graft Rejection (CGR) 

This is also called chronic transplant rejection (CTR). The allograft function is lost several months to years after grafting. Although the graft may still be in place, graft function loss is due to persisting immune system attacks on the allo-MHC. CGR is mediated by humoral as well as cellular immunity. Although immunosuppressants and tissue-typing methods are helpful in the first-year post-graft, CGR is almost always not preventable. It appears to be fibrotic scarring in the grafted organs, although the specific histopathology image depends on the grafted organ [67].

*Mechanisms of Rejection in Tissue Transplantation* 

**Figure 3:**
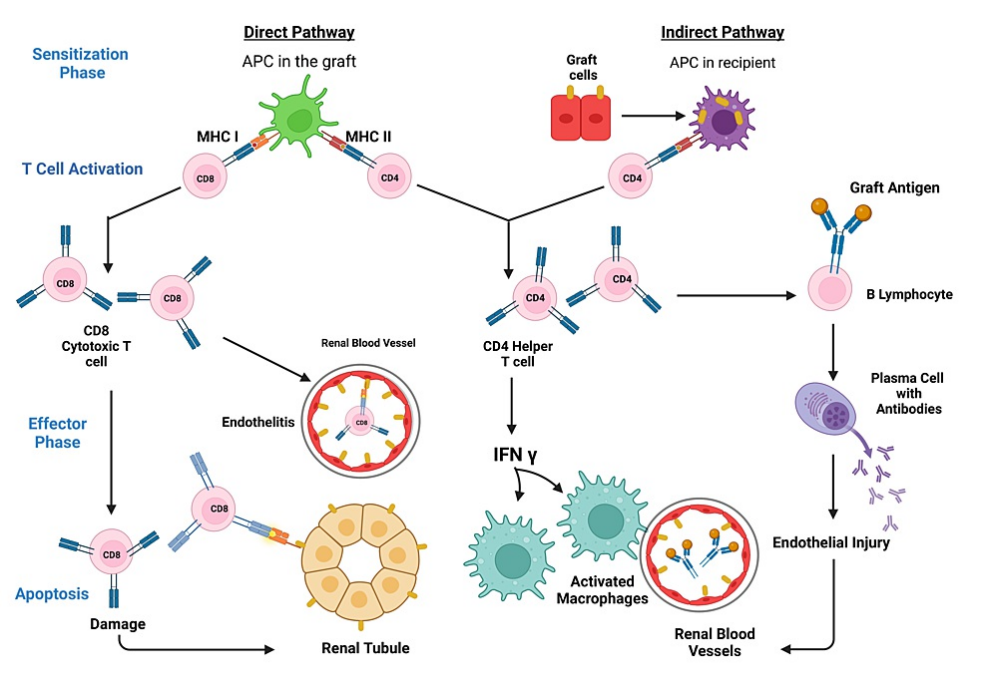
Clinical Stages of Graft Rejection. Notes: APC=Antigen Presenting Cell, MHC=Major Histocompatibity Complex, CD=Clusters of Diffrentiation, T Cell= A Subclass of Lymphocytes. This figure has been developed using Biorender [https://biorender.com/] License Number: DA24ILUA8K. Image Credit: Susmita Sinha.

The immunological reaction to the grafted organ is both lymphocyte and antibody-mediated. Nevertheless, the central player in transplant rejection is the T cell/lymphocyte [68]. There are 2 phases in transplant rejection (Figure 3): a sensitization phase and an effector phase [50].

Sensitization Phase 

Here, through their receptors, the helper (CD4) and cytotoxic (CD8) T-cells can identify the alloantigen displayed on the donor/foreign transplant cells. Antigen recognition begins with the T-cell receptor cross-talk with the antigen expressed by MHC molecules, followed by the costimulatory receptor/ligand cross-talk with the T-cell/APC surface [69]. One of the several costimulatory pathways involved in the sensitization phase is the communication between the T-cell surface CD28 with its APC surface ligands, B7-1 or B7-2 (referred to as CD80 or CD86, respectively) [70]. Also, CD8-associated antigen-4 (CTLA4) binds to B7-1 or B7-2 ligands to provide signals that cancel effects. CD40 and its ligand CD40L (CD154) equally serve for co-stimulation in this phase. Typically, the two convolutions of the MHC molecules form a peptide-binding groove to take up the peptides of normal cellular proteins origin. Thymic or central and peripheral tolerance mechanisms swing into action to ensure that the formed self-peptide-MHC complexes are unrecognizable by the T-cells, suppressing any possible autoimmune responses [71]. The two distinct but interrelated pathways of allorecognition are the direct and indirect pathways, generating specific groups of allospecific T-cell clones. 

Direct Pathway/Mechanism 

The direct mechanism is the primary pathway seen in early immunological response. Here, the host/recipient T-cells identify whole allo-MHC molecules found superficially on the donor or stimulator cell. The recipient T-cells see allo-MHC molecule + allo-peptide as having the self-MHC + non-self-peptide shape and determine the donating tissue as non-self [50,72]. 

The grafted organ has an undefined number of passenger APCs that appear as dendritic cells occupying the interstices with intensely populated allo-MHC molecules. These can activate the recipient's T cells directly. When the allogeneic or donor cells interact with the T-cells, the T-cells proliferate profusely in comparison with the clone populations that target antigens displayed by auto-APC. This mechanism is suggested in acute allorejection [73]. 

Indirect Pathway/Mechanism 

T-cells identify refined alloantigens displayed as peptides by auto-APCs. Then, epitope switching or spreading in which T cells proliferate to a more variable repository, such as initially immunologically dormant peptides [74]. Ali et al. [75] demonstrated that the connection of self-MHC + allopeptide-primed T cells with acute vascular type rejection is partially modulated via the production of augmented alloantibody. In contrast, chronic allograft vasculopathy is modulated by primed T cells.

Molecular Interactions in T-lymphocyte Activation

During T-lymphocyte (T-cell) stimulation, inositol phospholipid molecules in the cell membrane are added to water molecules to form diacylglycerol (DAG) and IP3 [75], resulting in the influx of Ca2+ into the cytoplasm [76]. This provokes a series of events that form calcium-calmodulin complexes, stimulation of several kinases, protein phosphatase IIB or calcineurin, and calcineurin dephosphorylates cytoplasmic, nuclear factor of stimulated T cells (NFAT) and thus causing NFAT to relocate from the cytoplasm into the nucleus. In the nucleus, NFAT combines with the Interleukin-2 promoter sequence to activate the synthesis of Interleukin (IL)-2 mRNA from DNA [77]. Several other events also take place within the T cell, such as protein kinase C (PKC) stimulation by diacylglycerol (DAG) and stimulation of nuclear factor kappa B (NFkB) [76,78]. 

Effector Phase 

The effector phase is the second phase in organ transplant rejection that involves alloantigen-dependent and independent factors. Reduced blood flow initially induces a nonspecific inflammatory reaction, leading to increased antigen presentation to T cells due to the upregulated expression of adhesion molecules [79]. Also, intact soluble MHC molecules are liberated to stimulate the indirect allorecognition pathway [80].

Within the first few weeks after tissue transplant, several T lymphocytes and their derived cytokines like IL-2 and IFN-γ are generated. Later, RANTES (Regulated on Activation, Normal T Cells expressed and secreted), MCP-1, and IP-10 are produced, leading to the influx of many macrophages into the allograft. The effector phase is also marked by upregulation of Interleukin-6, Tumor Necrotic Factor-α, inducible nitric oxide synthase (iNOS), and growth factors leading to rapid multiplication of smooth muscles, thickening of the inner lining of lymph and blood vessels, interstitial fibrotic scarring and, in the case of the kidney, scarring or hardening of the glomeruli [57,58]. MHC class II molecules, costimulatory molecules, and adhesion molecules are expressed following the stimulation of the endothelial cells by T lymphocytes-derived cytokines and macrophages [81,82]. 

Apoptosis 

Apoptosis is the last stage involved in tissue rejection. It is the usual mechanism for the cell-killing processes leading to the programmed death of the target cell [82]. Post-stimulation of the cytotoxic T lymphocytes involves the generation of cytotoxic granules containing (a) serine proteases (called granzymes) that induce programmed cell death and (b) pore-forming cytolytic proteins (perforin) [82,83]. The cytotoxic granules join the effector cell membrane during target cell recognition and arrangement and liberate its content into the immune synapse. The granzymes insert into the target cell cytoplasm to induce programmed cell death (apoptosis). This is the common cause of apoptosis in allograft rejection [83]. The fas-dependent pathway is another important pathway CD8+ can employ to achieve cytolysis and apoptosis and limit T-lymphocytes' rapid multiplication in response to stimulations to antigens. Cell-mediated cytotoxicity plays active functions in acute allograft rejection [84,85].

Role of Natural Immunity in Graft Rejection

**Figure 4:**
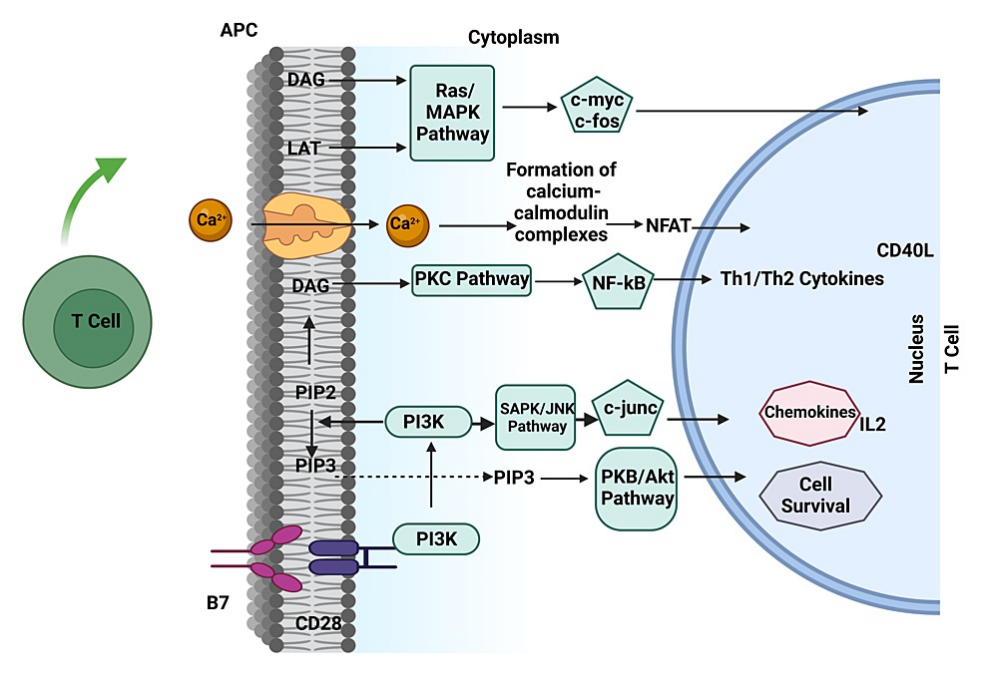
Role of Natural Immunity in Graft Rejection. Notes: APC=Antigen Presenting Cell, DAG=Diacyl Glycerol, LAT=Linker for Activation of T-Cell, MAPK=Mitogen-Activated Protein Kinase, RAS=Rat Sarcoma, PKC=Protein Kinase C, SAPK=Stress Activated Protein Kinasse, JNK=c-Jun N-terminal Kinase, CD=Clusters of Differentiation, PIP=Phosphatidyl Inositol Phosphate, PKB=Protein Kinase B, NFκβ= Nuclear Factor kappa beta, Th=T hepler cells, IL=Interleukin. This figure has been developed using Biorender [https://biorender.com/] license number: VY24J04THQ. Image Credit: Susmita Sinha.

The T-lymphocytes unarguably play an essential role during acute organ rejection (Figure 4). However, the increase in pro-inflammatory mediators in the allograft occurs before the T lymphocytes response is seen as an innate response to tissue injury and does not depend on the acquired immunity [86,87]. 

Even though natural mechanisms alone do not lead to transplant rejection, they are necessary for optimal acquired immunological reactions to the transplant. They are also vital in resistance to tolerance induction [88,89]. Although essential in particular disease management, cutting off the natural immune responses most assuredly impacts tissue grafting [86].

Natural Killer (NK) Cells

NK cells can discriminate between allogeneic cells and self and have robust cytolytic effector mechanisms to establish as much effector response as possible, even without previous immune sensitization [90]. Unlike lymphocytes, NK cells can be stimulated even without MHC molecules. This is possible due to the several NK inhibitory receptors produced by specialized alleles of MHC class I antigens on cell surfaces. NK cells are also equipped with stimulatory receptors activated by antigens on non-self-cells. NK cells also assist CD28+ host T lymphocytes and encourage allograft rejection [91]. NK cells have been identified to play an active role in chronic and acute rejection of solid organ grafts [92]. In addition, they also modulate allograft outcomes of the heart.

Neutrophils

Because of their number and high motility, neutrophils are the prime white blood cells to migrate to grafted organs and have been recognized as potent markers of transplant injury [93]. 

The release from dead cells upregulates the stimulation and subsequent neutrophil infiltration into grafted tissues, and the extracellular matrix is of damage-associated molecular patterns (DAMPs) [94]. DAMPs also trigger the generation of inflammatory cytokines by activating pattern recognition receptors (PRRs) on macrophages. These inflammatory cytokines include ELR+ CXC chemokines and IL-1β, which play some critical functions in neutrophil recruitment [95]. In addition, neutrophils also exhibit PRRs. When activated by DAMPs, they evoke a series of events, including; the production and release of reactive oxygen species (ROS) and hydrolyzing enzymes that aggravate damage to transplanted organs/tissues. 

Although not counted among the professional antigen-presenting cells (APCs), neutrophils can migrate from peripheral sites to transport their antigens to lymph nodes [96]. They can also trigger T-cells differentiation by an exhibition of MHC and costimulatory molecules [97]. Neutrophils are also known to contribute to clearing inflammation and start the production of anti-inflammatory substances among other myeloid cells [98,99].

Macrophages

These are highly motile, naturally trained immune cells capable of detecting, ingesting, and destroying disease-causing and other harmful particles. They constitute most parts of host defense and tissue homeostasis mechanisms and initiate the development of other immune cells [100]. Tissue macrophages are localized inside tissues, while blood macrophages originate from the monocytes that circulate in the blood and develop into macrophages in the bone marrow.

They are pivotal in the mediation of transplant immunopathology. Apart from mobilizing first-line defense against pathogenic organisms and functioning as APCs, they equally censure allografts as non-self-entity and encourage transplant loss by a similar mechanism [101,102].

Macrophages are implicated in ischemia/reperfusion injury (IRI), the alloimmune response, and acute graft rejection [103,104]. Macrophage mobilization happens immediately after reperfusion during organ grafting, and copious amounts of pro-inflammatory cytokines are generated to destroy the tissue [105,106]. Macrophages may also trigger graft rejection by activating acquired alloimmune reactions. They also furnish costimulatory signals that ease and augment the stimulation of T lymphocytes [101]. Transplant injury could be alleviated and graft survival prolonged if macrophages are deleted or inhibited [107]. Both clinical and animal studies demonstrated some positive correlation between allograft rejection and macrophage infiltration [108,109]. Also, in B cell-mediated rejection, there is demonstrable infiltration of macrophages and monocytes [110,111]. 

Graft Tolerance and Minimizing Rejection 

Tissue/organ graft is recommended for end-stage tissue/organ failure patients. The clinical practice's goal and challenge are striking a balance between the allogeneic immune response, the unwanted consequences of the immunosuppressants, potentially fatal infection, malignancies, organ toxic­ity, hypertension, and diabetes. Mitigating long-term immunosuppression through immunologic tolerance is highly recommended to ensure long-term patient and allotransplant survival. That graft recipients enjoy a better quality of life and improved life expectancy [8]. Transplant tolerance conserves stable allotransplant functions without immunosuppressive treatment [8]. Although rejection cannot be ruled out completely, some immunological tolerance to the grafted tissue does occur.

Some hypotheses on the development of transplant tolerance include adverse selection in the form of clonal deletion, absence of the normal immunological reaction to a particular antigen or allergen in donor-specific T and B cells, and formation of immune cells that blocks the actions of some other types of lymphocytes, or circumstances that decrease the immunological response against the transplanted organ and lingering dendritic cells (in the organ recipient) that are from an organ donor and which ensure immune-mediated chimeric state between the grafted organ and its recipient.

*Regulatory T Lymphocytes in Graft Tolerance* 

Ensuring allograft tolerance has become an ideal treatment goal in clinical transplant practice. Mitigating immunological reactions in allotransplantation and suppressing infections and tumor formation are significant hurdles in transplant practice. Although immunosuppressants effectively suppress acute rejection [112], currently utilized options cannot ensure that the recipient's immune system responds to antigens except those from donor alloantigens after transplantation [113,114]. 

**Figure 5:**
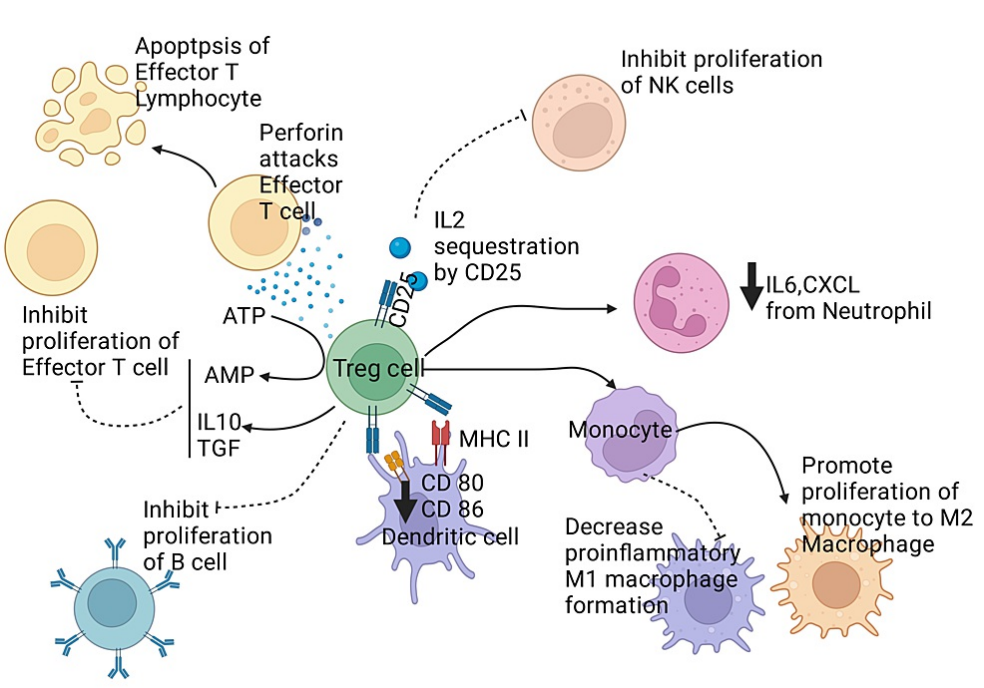
Illustrating the regulatory effect of T regulatory cells on the immune system. Treg cells release anti-inflammatory cytokines like IL10 and TGFβ and also convert ATP to AMP, which together inhibits the proliferation of effector T lymphocytes. Treg cells release perforin that attacks effector T cells and causes their apoptosis. CD25 expression from Treg cells causes sequestration of IL 2 and decreases the proliferation of Natural Killer cells (NK cells). Treg cells also directly inhibit the proliferation of B lymphocytes and reduce the expression of CD 80 and CD 86. Treg also promotes the differentiation of monocyte to M2 macrophages and suppresses the conversion of monocyte to M1 macrophages, which is pro-inflammatory. Treg also causes neutrophils to reduce the secretion of IL 6 and CXCL. Notes: Treg cell: T regulatory cell. NK cell: Natural killer cell. IL: Interleukin. TGF: Transforming Growth Factor. CXCL: CXC chemokine Ligand., ATP: Adenosine Triphosphate, AMP: Adenosine Monophosphate, CD: Clusters of differentiation, T Cell: Subclass of Lymphocytes, IL: Interleukin. This figure has been developed using BioRender [https://biorender.com/] License Number: PL24IU7VJY. Image Credit: Rahnuma Ahmad

Regulatory T cells (Tregs) refer to the specialized subset of T lymphocytes processing immunological reactions and ensuring homeostasis and self-tolerance. They suppress T lymphocytes' rapid multiplication and stimulation by cell-to-cell contact [115], modulate hyper-immune responses to non-self-antigens, and uphold self-tolerance [Figure 5] [115,116].

Pellerin et al. [117] suggest that Tregs are important in ensuring allograft tolerance. Treatments targeting Treg function and survival are novel options for ensuring immuno-tolerance in patients with organ transplants. CD25 and MHC class II expressions are the two important Tregs markers [118]. It has been demonstrated that successful allografting in humans is linked to a robust CD4+CD25+ Tregs population [119]. CD25+CD4+FOXP3+ regulatory T cells function to modulate immunological reactions to alloantigens and prevent rejection in-vivo [120]. Naturally occurring CD25+CD4+FOXP3+ regulatory T cells are produced as separate subsets during the differentiation of T lymphocytes in the thymus [121]. During organ grafting, CD25+CD4+FOXP3+ regulatory T cells (phenotypically and physiologically related to those derived from the thymus) may be triggered either in-vivo or ex-vivo alloantigen exposure [122]. The mouse model has also demonstrated similar regulatory T-cell functions [123].

Innate Immune Cells in Transplantation Tolerance 

Monocytes and Macrophages: Monocytes are blood phagocytes that form macrophages - the tissue-resident dendritic cells (DCs). Macrophages can modulate acquired immune responses and exhibit pro- or anti-inflammatory effects [124]. It has been previously stated that macrophages can contribute to allotransplant rejection via several mechanisms. However, evidence suggests they are also implicated in transplant tolerance in the adoptive transfer of regulatory macrophages (Mregs) [125,126]. These Mregs can inhibit the alloactivation of T lymphocytes via iNOS generation and function as critical mediators of transplant tolerance [126]. They are crucial in the induction of immuno-tolerance and have associated therapeutic involvement in tissue grafting [127].

Neutrophils: Neutrophils involved in programmed cell death (apoptosis) are also able to modulate inflammation by releasing Arginase-1 (a metabolic suppressor of T lymphocyte stimulation) and shedding microvesicles that bear anti-inflammatory mediators [128,129]. A unique neutrophil subset through matrix metallopeptidase-9 (MMP-9) expression is required for optimal reperfusion of grafted islets [129]. 

Natural Killer Cells: Administration of anti-CD28 monoclonal antibodies causes NK cells to enhance tolerance during kidney allotransplant by inhibiting pro-inflammatory immunity [130-133]. López-Botet et al. [134] posited that the pathway of tolerance induction by NK cells depends on the nature of the graft or the immunosuppressant therapy. Distinct subpopulations of NK cells can induce tolerance through specific pathways, such as toxicity of the white blood cell or/and cytokine release. This can be observed during chronic inflammation or infection. Here, NK cells are triggered, on exposure to IL-12, to secrete IL-10 [135]. IL-10 cytokine secretion by NK cells ensures that the fetus is not rejected by maternal allospecific T lymphocytes and inhibits inflammatory responses in the brain, spinal cord, and eye [136]. NK cells indirectly also trigger regulatory T lymphocytes in anterior chamber-acquired immune deviation (ACAID), leading to a generalized antigen-specific immune digression in the body [114]. The modulation of homeostatic CD8+ effector memory (TEM) enlargement by NK cells was perforin-independent, possibly moderated through competition for IL-15 cytokine [137]. NK cells can modulate the generation of tolerance by several pathways because of their cytolytic actions, cytokinogenesis, and capacity to compete for stimulation with cells aggressive toward "other" cells [138]. Depending on the nature of the graft and the recipient's alloimmune reactions, distinct NK cell subpopulations and pathways may be involved in tolerance initiation [139].

Cross-Matching and Use of Immunosuppressants to Mitigate Graft Rejection

Cross-matching is vital in the workup towards tissue transplantation as a lack of data on compatibilities between donor and recipient will result in a futile outcome. When a positive cross-match is obtained on testing, it implies a hyperacute rejection is a potential outcome in any recipient of such graft. The rejection is usually due to the presence of donor-specific antibodies (DSAbs) in the recipient's serum performed against one or several human leukocyte antigens (HLA) [140]. Despite their roles in graft rejection, the HLA proteins are important because they can help the immune cells differentiate themselves from non-self-proteins, preventing bodily harm. In addition, the variations in the HLA genes are numerous, leading to complexities in the immunology of transplants [141]. Pregnancy, blood transfusion, and previous transplantation are significant ways DSAbs usually develop [142]. While there are a couple of cross-matching techniques available, the occurrence of high graft loss despite negative cross-matches in high-risk patients caused a need for the development of more sensitive cross-matching methods [141], such as the enzyme-linked immunosorbent assay (ELISA) and Bead-based fluorescent assays [142]. One of the most straightforward techniques for cross-matching, as seen in the Complement-dependent cytotoxicity cross-matching, involves preparing a mix of the recipient's serum with T or B cells (T and B lymphocytes) from the donor with the addition of a complement. The presence of lysis and its proportion indicates whether the cross-match is assigned a weakly, moderately, or strongly positive grade [140]. From the preceding, the role of immunosuppressants in helping to mitigate graft rejection becomes clear. Research on immunosuppressive agents has increased steadily over the decades. The corticosteroids were first employed as far back as 1950, before the advent of antiproliferative agents such as azathioprine [143].

**Figure 6:**
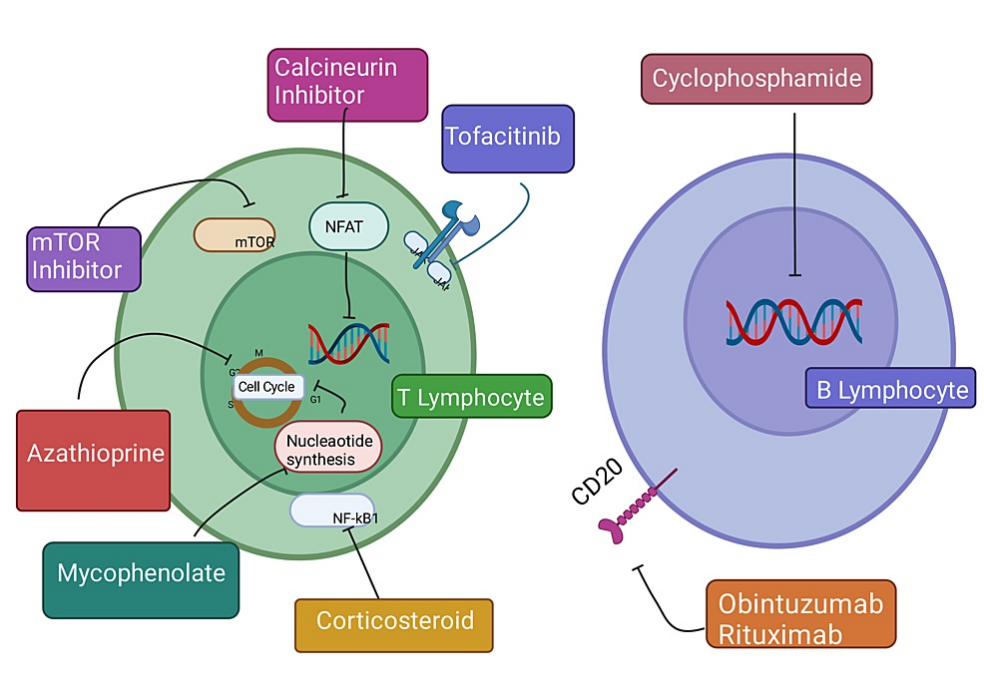
Showing the mechanism of different immunosuppressive drugs on T and B lymphocytes. Drugs inhibit specific pathways, cell cycle, and DNA synthesis by inhibiting mTOR, NFkB, NAFT, and JAK, which decreases lymphocyte activation and proliferation and promotes graft tolerance. mTOR: mammalian target of rapamycin. NFkB: Nuclear Factor kB. NAFT: Nuclear factor of activated T cells. JAK: Janus Kinase. This figure has been developed using Biorender [https://biorender.com/] license number: RC24IZS47Z. Image Credit: Rahnuma Ahmad

Cyclosporine A and tacrolimus, both calcineurin inhibitors, are the primary agents used around the globe. Other approved agents are sirolimus, mycophenolate mofetil, and belatacept, which were approved in the last decade by the Food and Drug Administration (FDA) [144]. There are many ongoing clinical trials for novel immunosuppressive agents with intended clinical relevance in organ transplants. Tocilizumab, fingolimod, and sotrastaurin are some current agents being investigated [143]. The alleviation of graft rejection through immunosuppressants could be through induction or maintenance therapy. The final aim of all agents in use is to diminish immune response to promote graft tolerance and suppress the effects of any positive cross-match, especially for sensitized patients. Blockade of T-cell activation, induction of apoptosis, prevention of T-cell proliferation, and inhibition of B lymphocyte differentiation into antibody-producing cells are common mechanisms of action of immunosuppressive drugs [Figure 6] [144].

The invention of an individualized treatment plan for organ recipients and the discovery of those agents which would reduce toxicity and side effects and increase therapeutic efficacy in graft tolerance are the properties expected of future immunosuppressive agents [143].

Regenerative Medicine and Tissue Engineering

Tissue engineering, as a field, seeks to understand and explore bio-substitutes for the restoration, maintenance, and improvement of the physiology of human tissues. In contrast, regenerative medicine as a field in health science seeks to understand and explore the processes involved in substituting, devising, or restoring mammalian cells, tissues, or organs to restore normal physiology. Tissue engineering and regenerative medicine (TERM) share many similar intended outcomes, leading to the coining of the acronym "TERM" to represent the two fields [145]. TERM is intended to help solve the significant problems with traditional transplantation: shortages in organ donors and immunologically engineered graft rejection [146].

Three key elements are necessary for the science and art of tissue engineering: scaffolds that serve as the extracellular matrix, cell sources, and a stimulus that could be in the form of growth factors [147]. While the scaffolds are mainly biodegradable materials, the cell samples could be obtained from tissues to be regenerated or, most recently, are usually stem cells (hematopoietic stem cells, embryonic stem cells, induced pluripotent stem cells, etc.). Growth factors will help in vascularization and cell differentiation [145]. Furthermore, in TERM, cells could be obtained from the same individual (autologous) or a different person (allogeneic). Xenogenic cells have also been experimented with, which, alongside allogeneic cells, can elicit immune reactions, resulting in a need for immunosuppressants [146].

There are variations in the regenerative capacities of different human tissues and organs, with the cornea and cartilage showing very limited or no regenerative abilities and the lung and liver having more abilities [146]. This notwithstanding, a vast amount of research has been done in tissue engineering in recent decades. However, they have yet to yield the desired bench-to-bed outcomes, especially in bone tissue engineering. In bone tissue engineering, this is primarily due to unsuccessful clinical trials, which are attributed partly to the manufacturing and designing ideal scaffolds [148]. Spinal cord injury is another infirmity requiring the innovation provided by TERM. Salgado et al. [145] hydrogels have been adequately researched to employ tissue engineering techniques to deliver human neural stem cells. 3D bioprinting has been a way of making better scaffolds because it allows biomaterials to integrate well into a patient's tissue and promote vascularization [146].

Future Perspectives

This review suggests the need for more advancement in research toward fighting tissue rejection and improving tolerance. It points to the multifaceted role of the immune cells in the concepts of graft rejection. Understanding the molecular biology of tissue transplantation facilitates the identification of the different proteins and pathways involved. This would enhance these proteins' genetic engineering and production in commercial quantities for prophylactic and therapeutic purposes. Also, the design of novel proteins through quantum computing can be possible at the proteomic dimension.

## Conclusions

Tissue transplantation is still a relevant area in medicine with the potential for more breakthroughs if the hindering challenges are overcome. Even when improved with genetic manipulations, xenotransplantation faces ethical and rejection concerns. The T lymphocytes involved in the sensitization and effector phases of tissue rejection are central to the immunology of tissue graft rejection. However, the regulatory Tregs are necessary alongside the regulatory macrophages to fight rejection and promote tolerance.
